# Spiritual Care in General Practice: Rushing in or Fearing to Tread? An Integrative Review of Qualitative Literature

**DOI:** 10.1007/s10943-018-0581-7

**Published:** 2018-02-23

**Authors:** Alistair Appleby, Philip Wilson, John Swinton

**Affiliations:** 1 Aviemore Medical Practice, Muirton, Aviemore, Inverness-Shire PH22 1SY Scotland, UK; 2 Centre for Rural Health, Centre for Health Science, University of Aberdeen, Old Perth Road, Inverness, IV2 3JH Scotland, UK; 3 School of Divinity, History and Philosophy, King’s College, University of Aberdeen, Aberdeen, AB24 3UB Scotland, UK

**Keywords:** General practice, Primary health care, Spirituality, Religion

## Abstract

Guidance for medical staff reminds employees of the responsibility to deliver spiritual care in its broadest sense, respecting the dignity, humanity, individuality and diversity of the people whose cultures, faiths and beliefs coexist in society. This is no small or simple task, and although GPs (family practitioners) have been encouraged to deliver spiritual care, we suggest this is proving to be challenging and needs further careful debate. This literature review critiques and analyses existing studies and points to four categories of attitude to spiritual care, and two related but distinct concepts of spirituality in use by GPs. Our aims were to search for, summarise and critique the qualitative literature regarding general practitioners’ views on spirituality and their role in relation to spiritual care. An integrative review was made by a multidisciplinary team using a critical realism framework. We searched seven databases and completed thematic and matrix analyses of the qualitative literature. A number of good-quality studies exist and show that some but not all GPs are willing to offer spiritual care. Four patterns of attitude towards delivering spiritual care emerge from the studies which indicate different levels of engagement with spiritual care: embracing, pragmatic, guarded and rejecting. Further research is needed to identify whether these four views are fixed or fluid, whether training in spiritual care modifies these and whether they relate to patterns of care in practice, or patient outcomes. The authors suggest that some of the difference in viewpoint relate to the lack of clear philosophical framework. The authors suggest *critical realism* as having potential to facilitate interdisciplinary research and create clearer concepts of spiritual care for GPs.

## Introduction

Qualitative and quantitative research studies have demonstrated that there are likely to be associations between certain religious and spiritual variables and health outcomes (Ellison and Levin 1998; Koenig 2011). These relationships may not be simple, and many of the studies are methodologically imperfect, but as more sophisticated research proceeds, these associations have persisted though there is some discussion about causality (Sloan 2006).

General practitioners in the UK more widely have been encouraged to think about incorporating spiritual care into their provision for patients (NHS Education for Scotland 2009). This advice to attend to a spiritual dimension of care specifically includes but is not confined to circumstances that include palliative care (Puchalski et al. 2009) and mental health care (Cook 2013). Interest in spirituality and health may reflect a wider discussion about the role of the biomedical model, and in examining what the humanities can bring to complex patient care problems (Misselbrook 2015). However, there is limited evidence that GPs are delivering effective spiritual care in any explicit way, or in one that effects outcomes.

Research to date has concluded that while in general most GPs see a role for themselves in spiritual care (Murray et al. 2003), this is not universal (Vermandere et al. 2011). The reservations of those who have not embraced this concept may contain insights that need to be more fully understood. We sought to clarify this situation via an integrative review: a form of research that reviews, critiques, and synthesises representative literature in an integrated way such that new frameworks and perspectives on the topic are generated (Torraco 2005).

We referred to a consensus definition of spirituality (Puchalski et al. 2009). This is thought to provide a set of parameters which has clinical usefulness in discussions around spirituality:

*Spirituality* The aspect of humanity that refers to the way individuals seek and express meaning and purpose and the way they experience their connectedness to the moment, to self, to others, to nature, and to the significant or sacred.

*Religion* We formed a definition of religion with reference to NHS spiritual care documentation (NHS Education for Scotland 2009) and by discussion to consensus. The definition adopted was “The characteristic beliefs and practices of a community of faith with particular reference to the worship of a divine being”.

### Aims of the Study

1. To summarise existing research related to general practitioners’ perceptions of spirituality, religion and spiritual care.
2. To explore whether a robust and useful concept of spirituality exists in primary care.
3. To identify gaps in the current literature and to suggest future research agendas.

## Methods

Our interdisciplinary team included academic staff of the University of Aberdeen’s rural health and theology departments and included researchers with both theist and atheist belief. We used basic critical realism as the philosophical framework for the study. Critical realism has the advantage of being able to hold the weight of discussion between the humanities and sciences without diminishing either (Roberts 2014). Critical realism views science neither purely within the limitations of Popper/Hempel terms, where knowledge can only be gained by experiment, nor in relativist constructivist terms where nothing can be absolutely true or real except in relation to the experience of a particular observer. Critical realism describes an objective reality, but one which can only be understood incompletely due to the limitations of the human processes by which it can be investigated (Bhaskar 2008). It has proven usefulness in social science research (Danermark et al. 1997).

We made reference to the PRISMA guidelines (Moher et al. 2009) and to the HTA guidance (Murphy et al. 1998) in creating a framework for the search strategy. We used PRISMA to create a clear rationale for the study, defining eligibility criteria, information sources and study selection. We followed the HTA guidance on synthesising qualitative data at this stage of the review, and we used three methods of study identification:

1. Formal electronic database search—2001 studies.
2. Cross-referencing to bibliographies of key papers—2 studies.
3. Expert peer review to ensure no papers were missed. No studies.

References and papers were sought from seven databases, using MESH headings and expanded terms and included both clinical scientific and social science/religion databases (e.g. ATLA). Each database was individually searched using a custom search, and all search results were available for inspection by the three reviewers.

### Inclusion/Exclusion Criteria

Studies were included where they:

1. Have a substantial focus on the research questions, i.e. investigated perceptions of spirituality as it related to the adopted definitions.
2. Presented original data
3. Related to general practitioners/primary care doctors
4. Were published, in English, in the last 30 years
5. Were published in peer review journals

Studies were excluded where they:

1. Did not relate to the chosen definition of spirituality.
2. Were individual case reports or studies involving the opinion of five, or fewer general practitioners.
  After reading full manuscripts of the retrieved studies, and in accordance with the HTA standards for qualitative research review, we decided also to exclude papers which:
3. Studied a heterogeneous group of carers or professionals, including but not confined to general practitioners, unless the views of the latter could be clearly differentiated.
4. Papers where the focus was on complementary therapies, case reports, and reports and studies of “faith healing” (Table 1).

**Table 1:** Search strategy for Ovid-based Databases

1. Family practice/or general practice/
2. Primary health care/
3. GP.tw.
4. GPs.tw.
5. General practitioner$.tw.
6. Primary care.tw.
7. Primary health care.tw.
8. General Practitioners/
9. Exp Religion/
10. Exp Spirituality/
11. Spiritual$.tw.
12. Pastoral care/
13. Perception/
14. Perception$.tw.
15. Attitude/
16. “Attitude of health personnel”/
17. Experience$.tw.
18. Understanding.tw.
19. Awareness.tw.
20. Opinion$.tw.
21. Idea$.tw.
22. Observation$.tw.
23. Thought$.tw.
24. 1 or 2 or 3 or 4 or 5 or 6 or 7 or 8
25. 9 or 10 or 11 or 12
26. 13 or 14 or 15 or 16 or 17 or 18 or 19 or 20 or 21 or 22 or 23
27. 24 and 25 and 26
28. limit 27 to (English language and humans)

Quality appraisal of studies:

We chose to qualitatively analyse the quality of studies to moderate the effect on our synthesis rather than to exclude certain studies (Gough et al. 2012).

Data extraction and analysis:

We performed a matrix analysis as described by Miles and Huberman (1994) as a way of highlighting and contrasting data from different studies to bring out unique insights and perspectives in relation to the way the data is configured.

In addition, we performed a formal grounded theory thematic analysis of participants’ views using QSR NVIVO 10 (Glaser and Strauss 1967). The research team assumed responsibility for the authenticity and validity of this process, which occurred through regular cross-disciplinary meetings.

### Synthesis of the Data

**Fig. 1:**
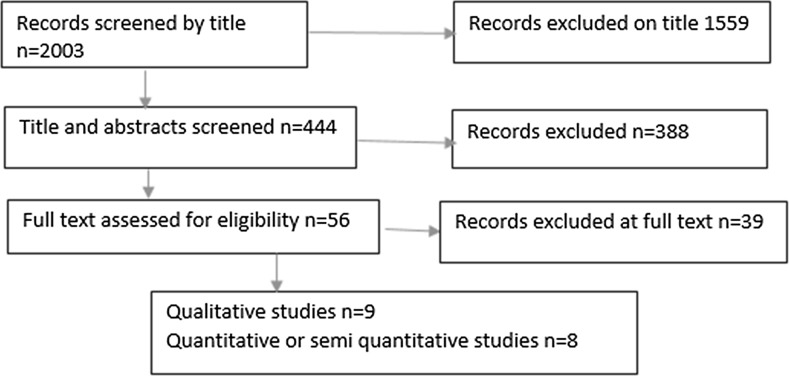
PRISMA-based flowchart shows inclusion and exclusion of studies at each stage

Experience and opinion from diverse cultures cannot simply be added together and merged conceptually (aggregation) without great care (Pope et al. 2007). In common with principles of ethnographic research, we chose only to perform limited aggregation and tried to preserve the context of the studies unless the concepts of spirituality were clearly analogous (Noblit and Hare 1988) (Fig. 1).

## Results

All of the nine qualitative studies had robust methods of recording, transcribing and analysing. One study did not have a prospective design and relied on video initially and later on field notes (Saba 1999). Two studies explicitly stated an overall philosophical framework (phenomenology), and eight had a robust description of their methodology of analysing data, grounded theory being the most common. Most studies did not offer a concept of spirituality to its participants, probably legitimately because GPs’ own concepts were often sought.

A total of 294 GPs’ opinions were investigated. Three studies asked more fundamental questions about practice, and spirituality arose as a significant theme in participants’ responses. These three began their focus on: listening in primary care and awareness of personal boundaries to care (Cocksedge and May 2009), managing palliative patients (Kelly et al. 2008) and on what GPs believe and value in their work (Saba 1999).

No studies made specific attempts to investigate GPs’ perceptions of *religion* or its relation to health. However, respondents frequently made reference to religion in their answers, consistent with the idea that religious orientation and practice functionally and conceptually are an aspect of, but not necessarily mandatory for, GPs’ understandings of spirituality. A minority of researchers chose to ascertain or record religious affiliations of participants. There are some theoretical reasons why asking religious affiliation may detract from the validity of interviewees’ subsequent opinions: for example, participants may feel they are representing the views of their faith community rather than their private view. The failure to ascertain religious affiliation could also be seen as a weakness in ascertaining diversity of the sample. Where they were stated, participants’ affiliations included: Atheist, Agnostic, Muslim, Seventh Day Adventist, Christian, Jewish, Unitarian, “Universalist with Buddhist leanings” and “none”. The majority of participants whose beliefs could be identified were Christian (Table 2).

**Table 2:** Table summarising the main characteristics of the qualitative studies

First author and title of study	Context and main focus	Sampling and participants	Spirituality concept	Methodology	Main results and conclusions	Barriers/facilitators
Cocksedge, S. Doctors’ perceptions of personal boundaries to primary care interactions: a qualitative investigation (Cocksedge and May 2009)	UK, 2009. The boundaries of GP care in relation to touch and spiritual care. Both these aspects of care had arisen as significant themes from a previous qualitative study by the authors	Semirural Northern English GPs. Convenience/geographical sample. 28 GPs in this area invited. 23 accepted	Author uses Murray definition 2003 “The needs and expectations that all humans have to find meaning, purpose and value in life”. Many respondents clearly connote religious affiliation with this and respond in relation to this	Qualitative: Recording, verbatim transcription, thematic analysis using constant comparative method (Strauss and Corbin 1998). Coding involved 2 researchers	Spiritual needs may need to be addressed but there are differences in perception of how often these present. Religiously affiliated GPs report seeing more and are more confident in dealing with this. Those without faith report less presentations of spiritual problems and may be less keen, or unwilling to address these. Three reported no instances of spiritual issues presenting in primary care	Personal lack of the awareness of spirituality may create functional boundaries to discussion with patients. One practitioner “acts” a religious perspective where it seems the patient might wish this
Craigie, F. C. Spiritual perspectives and practices of family physicians with an expressed interest in spirituality (Craigie et al. 1999)	USA 1999. Views of family practitioners on spirituality An open ended empirical enquiry in relation to patient encounters, personal practice and medical education	Intentional sampling of interest group. 12 word of mouth/snowballing invitations to PCPs “with a significant interest in discussing spirituality” Participants were heterogeneously religious	Not explicitly stated, implicitly existential, meaning based, sometimes religious	Qualitative: Convenience sample. Data collection to theoretical saturation. Qualitative interviews, transcriptions, 7 stage phenomenological analysis based on Colaizzi (1978)	Spirituality personally highly significant for the practice of medicine, Need to respect patients own beliefs. Participants report spirituality underpins the vocation of many of them and they see a vital goal of PCPs as encouragers of a patient’s own spirituality. Dialogue, respect and mentoring important aspects of spirituality teaching	Barrier: time. Facilitators; ongoing, genuine and respectful relationships. Comments about personal, clinical and organisational axes of spiritual care. Section on future research including” collecting stories about physicians experiences of spirituality”
Ellis, M.R. What do Family Physicians think about spirituality in clinical practice? (Ellis, Campbell, Detwiler-Breidenbach, and Hubbard 2002)	USA 2002 Views of spirituality and attitudes about provision of spiritual care	13 family practitioners from Missouri. Purposive sampling to locate diversity of age, sex, rurality and religious/non-religious background. Mostly Christian, some agnostic and atheist	Not defined but functionally dealt with as broad inclusive concept including and perhaps most closely related to Judeo-Christian ideas. Clearly includes religious belief but not exclusive to this	Qualitative: Semi-structured interviews with 13 Family Physicians. Audiotaping and use of “Ethnograph” textual analysis software. Preconceptions noted by researchers before analysis started to reduce bias. 6 stated Christian and 2 Agnostic or Atheist. Purposive sampling in regard to demographics and religious affiliation. Thematic analysis as per Miles and Huberman (1994)	Participants ranged from those who felt spiritual issues must be addressed as a scientific imperative, and those that felt its “primacy in life” justified this. Most PCPs saw a vital role of PCPs as encouragers of patients own spirituality but not necessarily as spiritual counsellors. One reported being opposed to addressing spiritual issues with patients because of issues about role definition and invasion of privacy. Some contexts more likely to prompt spiritual discussion, e.g. new diagnosis of a serious illness. Unique theme is modelling of spiritual maturity by the physician and that spirituality may be important for its own sake, not as a means to a health end	Family practitioners are more likely to address spiritual issues in terminal diagnosis, admission to intensive care and mental health. Concerns about imposing beliefs on patients. Physician and patient factors important. Physician factors include upbringing and culture, spiritual awareness, belief of failure to effect patient’s illness or lives
Grant, E. Spiritual issues and needs perspectives from patients with advanced cancer and non-malignant disease (Grant et al. 2004)	Scotland 2004. The attitudes and insights of GPs regarding spiritual care in the context of palliative care	Terminally ill patients and their GPs Likely to be but not stated to be similar to Scottish religious demography	“The needs and expectations which humans have to find meaning, purpose and value in their life”. Such needs can be specifically religious, but authors assume that even people who have no religious faith or are not members of an organized religion have belief systems that give their lives meaning and purpose”	Qualitative: Purposive sampling to ensure diversity in patients participants but not necessarily GP participants. In depth qualitative interviewing (Mays and Pope 1996). Taped, coded, analysed in iterative way	Spiritual care is important to patients in the palliative care context. GPs feel they lack time and skill to deliver this, though the paper shows that some are clearly delivering some components of religious care. Doctors who develop good relationships with patients may inadvertently provide spiritual care. Tentatively suggest lack of spiritual care may increase health care usage and propose mechanism for this. Patients can often meet their spiritual needs if this aspect is validated by professionals	Yes, see left box. A few GPs felt it would be inappropriate to raise such intimate issues
Kelly, B. General Practitioners’ experiences of the psychological aspects of care of the dying (Kelly et al. 2008)	Australia 2007. GPs experiences of the psychological aspects of caring for dying patients	A convenience sample of 15 doctors was recruited for the study at the point of referral of their patient to a hospice/home care specialist palliative care service	No definition offered—authors and participants definitions seem to have differed significantly	Qualitative: Interviews with a consultant psychiatrist as the researcher, audiotaping, thematic analysis	Participants connoted spirituality with religion and many did not think this was part of their role. These participants reported existential issues are some of the most difficult to broach. GPs’ reported leaving it to patients’ initiative to bring up prognostic or spiritual matters and implicated patients’ stoicism and wishes not to engage with these issues as limiting factor, rather than any personal characteristic of the GP	Predominantly barriers, no facilitators discovered or discussed
Murray, S. A. General practitioners and their possible role in providing spiritual care: A qualitative study (Murray et al. 2003)	Scotland 2003 GPs’ views of spirituality and attitudes to being spiritual care providers	Convenience sample: 40 GPs of patients with palliative care patients, their patients and their carers	Defined spiritual needs as the “needs and expectations that all human beings have to find meaning, purpose and value in life”	Qualitative interviews with 2 explicit research questions. Do family practitioners perceive a role in providing spiritual care and what might hinder them in assessing spiritual needs or providing spiritual care? Telephone interviews with experienced social scientist, taping, transcription, NVIVO thematic analysis	Most family practitioners have a high awareness of the spiritual needs of their dying patients and feel that they have a role in providing spiritual care but lack time and appropriate strategies to introduce this. These GPs conceptualise patients’ spiritual needs as broader than simply religious needs	Barriers—some GPs felt patients could be “the wrong type” of person for this approach. Time constraints
Olsen, M. M. Mind, body, and spirit (Olson, Sandor, Sierpina, Vanderpool, and Dayao 2006)	USA 2006. An exploration of family practitioners’’ beliefs and attitudes regarding integration of spirituality in patient care	Southwest US medical school. Convenience sample: 17 third-year family medicine residents	None explicitly offered. Implicitly existential//theistic/religiously pluralistic concepts of spirituality	Qualitative: Phenomenology/grounded theory (Strauss and Corbin 1990). Taping, transcription, coding, independent analysis multiple researchers	Family practitioner–patient relationships. Some report struggling with language and concepts to describe existential suffering. Concerns expressed to not try and infringe on other people’s personal beliefs and the possible misuse of medical power	Barriers such as initial reluctance, time constraints, ambiguity, and degrees to which some residents’ religious and spiritual orientations differ from those of patients
Saba, G. W. What do family physicians believe and value in their work? (Saba 1999)	USA,1999 For PCPs in training what core values are important to them in their work. Spirituality arises as a significant theme	Convenience sample or 143 Family medicine residents of a San Francisco hospital. Residents are ethnically a mixture of white, Hispanic and black Religious affiliation not recorded	Author does not adopt any position, but relates the definition and understandings of the participants, which are heterogeneous	Qualitative: Focus group with field notes, some videotaping. Group reflections on beliefs and values. Grounded theory related thematic analysis	Philosophical and religious values give meaning and moral direction to decisions that doctors make, and the decision to pursue medicine. Beliefs and values of residents about meaning, suffering and spirituality are essential to who they are and what they do as family practitioners	Not a concern of this paper
Vermandere M. GPs’ views concerning spirituality and the use of the FICA tool in palliative care in Flanders: A qualitative study (Vermandere et al. 2012)	Belgium 2012. Attitudes to use of a spiritual needs screening tool in palliative care	Convenience/geographical sample. 23 Belgian GPs in the vicinity of Leuven University	Uses Puchalski 2009 definition, as modified by the European Association of Palliative Care. multidimensional—existential, value based, religious/theistic	Qualitative: GPs chosen by location—Surrounding the Catholic hospital of Leuven, 12 researchers, 11 open ended questions. Verbatim recording and transcription. Line by line coding. Descriptive and interpretive themes developed	Opinions about the role of GPs in spiritual care were divided. Some reported this to be task for relatives or professional spiritual care providers. A majority felt spiritual care important but only half would initiate a discussion. PCP viewed the use of the FICA screening tool as a useful guide but should not be used prescriptively	Barriers: Lack of time, privacy or knowledge about the patients’ beliefs. Lack of spiritual education and of a shared spiritual language with the patient. Western societies discomfort with spiritual language and concern about disruption to relationship with patient

Thematic analysis of the studies showed several common themes emerging.

We identified four which related to GPs’ attitudes to being spiritual care providers. These were:

1. Embracing—GPs who accept and approve a role for GPs in spiritual care.
  “It’s an inherent part of the whole person, spirituality” GP 1. “If it’s unmet then that will impact on their overall health” GP 2 (Murray et al. 2003)
2. Pragmatic—GPs who accept a role if it is deemed to help or be wished by the patient.
  “Yes, I do see dealing with these as part of my role. But I generally consider that it would be up to him and his wife to raise them and they haven’t done so to date.”(Murray et al. 2003)
3. Guarded—GPs who have reservations about the role and might consider providing spiritual care with some provisos or in some limited contexts.
  “Everyone says ‘‘this only takes a few minutes.’’ If you keep adding that up it’s more than the 10 min that you have with a patient. It’s definitely important, but you can’t address it at every single visit.” (Olson et al. 2006)
4. Rejecting—GPs who are fundamentally opposed to the role, regardless of circumstance.
  “I think in the first place that it is not my personal responsibility to start up a conversation with the patient about ‘spiritual care’. So is that my role? Is that my task? Is that my duty? I don’t think so.” (Vermandere et al. 2012)

There are suggestions of a possible causal relationship of a GP’s attitude to providing spiritual care dependent on the personal belief of the GP.

“I do discuss it with some of my patients. I’ve got quite a lot of people from church who perhaps choose to see me rather than one of my partners. I would never push it down people’s throats, but if it’s obviously something they want to talk about, then I don’t feel uncomfortable because I have a faith of my own.” (Cocksedge and May 2009)

The author commented: “Of the three doctors who could not recall ever having had any consultations involving spiritual issues, two professed no personal faith”

There are also narratives that indicate that some GPs can, through awareness of their own personal assumptions and boundaries, modify and broaden their approach from their own beliefs or philosophical position to be more congruent with the patient or situation.

Two differently centred but overlapping concepts of spirituality were identified in the GP narratives.

1. Exocentric: relating to connection with an ultimate spiritual reality which is independent of the individual. This concept expresses a recognition that spirituality transcends the individual towards an identifiable “Other” in its locus and intention. The commonest expression of exocentric spirituality in the literature is theocentric: relating to connection with the divine or to an ultimate being, usually through religious affiliation or commitment. One respondent put it in this form, although it arose in many other forms.
  “Spirituality is seeing people as unique human beings whom God has created.” (Craigie et al. 1999)
2. Anthropocentric: Centred on the individual’s needs. A quest for personal or individual meaning, human connectedness, the fulfilment of a deep existential need.
  “[Spirituality is] a personal attitude to life, an outlook on life, … […] Spirituality is, to a certain extent, the time that you invest in the way you relate to things.” (Vermandere et al. 2012)

These concepts were not mutually exclusive, and participants often used *theocentric* and *anthropocentric* concepts of spirituality side by side. These concepts seem to be distinct in their focus, but used by many GPs without any sense of boundary between them.

“Religion is one of the forms, I think, in which spirituality can be expressed or defined. Thus I think it is a subset of the larger set we call “spirituality”. Uhh … beyond that, I don’t really see any other distinctions. But I think that you can perfectly well be spiritual in a non-religious way. That is also possible. And, yes, it seems to me that there are many different mixed forms of spirituality.” (Vermandere et al. 2012)

Another way of thinking about this would be to distinguish expressions of spirituality that have a concept of sacredness, (Pargament and Mahoney 2002) and those that do not.

## Discussion

Most studies use convenience sampling, some used geographical sampling and a few use theoretical or intentional sampling. Few authors made more than a very superficial attempt to describe the belief demographic of the patient population. This knowledge forms an important context in which GPs deliver spiritual care. Critical realism states that cause and effect in society are, at least in part, local and contextual, e.g. positive associations between health and spirituality in one context may not operate in others.

In collating studies, it is important to recognise the significant cultural and religious differences which exist between study locations, for example between the UK and the USA. The cultural acceptability of religious belief and the prevalence of religious affiliation vary in these contexts (“Religion—Ipsos Global Trends,” n.d.). Similarly, the perceived role of GPs in society and understandings of appropriate professional behaviour are likely to vary. Terms like “spirituality” and “religious” may have different usage or connotations in different countries and these differences are likely to be reflected in GPs perceptions.

Study participants seem frequently to make assumptions about the correlation between religious service attendance and the beliefs of their patients. In patient populations, differences in belief, commitment and practice are known not to fit into simple categorisations (Aisthorpe 2016; Levin and Meador 2012). Likewise, few GPs comment on the diversity of religious affiliation in their populations. With some exceptions, we suggest that where affiliation could be ascertained there was generally a higher focus on a Judeo-Christian perspective of spirituality both from the point of view of authorship and participants.

Most research teams were composed of clinicians although three included psychiatry, nursing or social science departments. Concern has been expressed from more than one perspective that research which treats religious experience and belief purely as a health variable are limited as they rely on a reductionist position and in this respect there are no multidisciplinary studies which include the humanities. We argue this may be due to the difficulty that clinical sciences and the humanities experience in dialogue. We propose from both the practical experience of our research team and from the arguments of critical realism that this difficulty arises from the use of incompatible epistemologies, ways of discerning truth. Critical realism allows for a middle-ground approach using a more flexible and robust philosophical framework. We propose a critical realism approach may promote more effective dialogue and reflects a more current approach to science.

### Implications for Future Research

Future studies which would be valuable might:

- Be conducted in multiethnic and religiously diverse environments or among GPs with a wider range of religious affiliation. Have stronger sampling strategies to ensure better saturation of data.
- Recognise that it may not be possible to easily aggregate research that is culturally diverse.
- Be conducted with explicit philosophical frameworks which support interdisciplinary discussion and are consistent with current thinking on the philosophy of science. We offer critical realism as a possible framework.
- Investigate GPs’ opinions regarding religion and spirituality as distinct, but related concepts.
- Investigate any possible causal associations between GPs’ personal beliefs and their perceptions of spirituality, their willingness to offer spiritual care, or the type of spiritual care they offer. Investigate to what extent GPs can show pragmatism or flexibility in delivering spiritual care, despite their own beliefs.
- Conduct research which tests the acceptability and outcomes of different models of spiritual care delivered in primary care

## Conclusions

The concept of spiritual care in general practice suggests potential (Best et al. 2015) but needs more careful debate. Critical realism may provide an intellectually persuasive and practical framework for these discussions and avoid polarised arguments.

There is a good deal in common with the concept of spirituality which GPs use in practice but we argue that two differently focussed but not mutually exclusive concepts of spirituality are represented by GPs: exocentric and anthropocentric.

At least four different types of attitude to providing spiritual care exist in the literature: embracing, pragmatic, guarded and rejecting. Further research is needed to investigate whether these attitudes are fixed or fluid. There is some evidence in the literature that these attitudes influence both the likelihood and the nature of spiritual care being delivered by the GP. It may be worth investigating whether training, or other characteristics such as pragmatism or patient centeredness, may modify these views in terms of delivery of spiritual care.

If it is thought desirable to encourage spiritual care in general practice, we will need to find common frameworks for these discussions and recognise and learn from our current diversity of views. Perhaps that will allow us to neither rush in, nor fear to tread.

**Table:** 

How this fits in: A modest number of studies from the USA, Europe, and Australia have described provisional but not universal acceptance of a spiritual care concept and role for GPs. This literature review critiques and analyses existing studies and points to four categories of attitude to spiritual care, and two related but distinct concepts of spirituality in use by GPs. The review recognises a need for further studies in a multi ethnic context and research with robust philosophical frameworks. The authors suggest *critical realism* as having potential to facilitate interdisciplinary research and create clearer concepts of spiritual care for GPs	
